# Paired Associative Stimulation Fails to Induce Plasticity in Freely Behaving Intact Rats

**DOI:** 10.1523/ENEURO.0396-19.2020

**Published:** 2020-03-19

**Authors:** Windsor Kwan-Chun Ting, Maxime Huot-Lavoie, Christian Ethier

**Affiliations:** Centre de Recherche CERVO, Département de psychiatrie et de neurosciences, Université Laval, Québec, Québec G1J 2G3, Canada

**Keywords:** Hebbian plasticity, motor cortex, paired associative stimulation, rodent models, spike timing-dependent plasticity, spinal cord

## Abstract

Paired associative stimulation (PAS) has been explored in humans as a noninvasive tool to drive plasticity and promote recovery after neurologic insult. A more thorough understanding of PAS-induced plasticity is needed to fully harness it as a clinical tool. Here, we tested the efficacy of PAS with multiple interstimulus intervals in an awake rat model to study the principles of associative plasticity. Using chronically implanted electrodes in motor cortex and forelimb, we explored PAS parameters to effectively drive plasticity. We assessed changes in corticomotor excitability using a closed-loop, EMG-controlled cortical stimulation paradigm. We tested 11 PAS intervals, chosen to force the coincidence of neuronal activity in the motor cortex and spinal cord of rats with timings relevant to the principles of Hebbian spike timing-dependent plasticity. However, despite a relatively large number of stimulus pairings (300), none of the tested intervals reliably changed corticospinal excitability relative to control conditions. Our results question PAS effectiveness under these conditions.

## Significance Statement

Paired associative stimulation (PAS) can be applied noninvasively to modulate corticomotor plasticity in humans. However, our understanding of how we can use paired stimuli to produce the greatest beneficial reshaping of corticomotor connections *in vivo* is still rudimentary. We completed a systematic study varying interstimulus intervals between cortical and muscle stimulation in a freely behaving rat PAS model, following the principles of spike timing-dependent plasticity (STDP). Crucially, our experiments have *not* demonstrated that the STDP model is effective *in vivo* using our PAS protocol. We discuss several other factors in addition to the interstimulus interval, which may play a larger role in driving plasticity, and potential ways that the field can approach future work.

## Introduction

### Spike timing as a driver of synaptic plasticity

**Figure 1.**
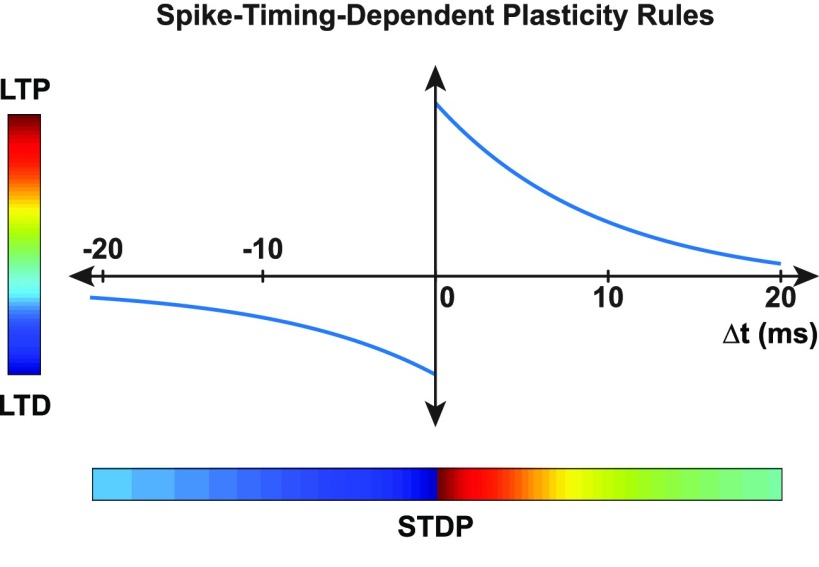
STDP rules. At the synaptic level, but to a lesser extent at the systems level, it has been demonstrated that the relative timing of activity between the presynaptic neuron and postsynaptic neuron is crucial for plasticity. When presynaptic activity repetitively occurs within several milliseconds prior to postsynaptic activity, LTP (red) is induced. When the timing is reversed, LTD (blue) is induced. The potential for LTP or LTD decreases as the time window between the presynaptic and postsynaptic activity at the synapse increases (Song et al., 2000).

Seminal studies on synaptic plasticity have led to the development of the spike timing-dependent plasticity (STDP) model (Markram et al., 1997; Bi and Poo, 1998), which is an extension of the Hebbian postulate (Hebb, 1949; Fig. 1). Whether synaptic potentiation [long-term potentiation (LTP) or long-term depression (LTD)] occurs is contingent on the pattern of firing activity in the presynaptic and postsynaptic neurons (Cooper, 2005). These concepts led to the development of stimulation-based neuromodulation methods aimed at conditioning cortical and spinal motor circuits to promote motor recovery after neurologic lesions.

However, the STDP hypothesis as it applies to larger circuits such as the corticomotor system is contingent on certain assumptions, one being that principles derived from *in vitro* studies at the synaptic level remain sound when applied to higher-level systems *in vivo*. Beyond the complexity of the anatomy of the system, ongoing patterns of neural activity (spontaneous or behavior related) may interfere with the fine-tuned firing patterns that STDP putatively requires, introducing variability into the equation. Hence, the field would benefit from more systematic study of STDP at the systems level in conjunction with ongoing neuronal activity.

### Noninvasive paired associative stimulation in humans

In humans, paired associative stimulation (PAS) using transcranial magnetic stimulation (TMS) and transcutaneous peripheral nerve stimulation is a noninvasive method used to modulate the excitability of corticomotor connections and to facilitate the recruitment of targeted muscles, based on Hebbian STDP principles. The first clear demonstration of PAS was designed to promote plasticity at the cortical level (Stefan et al., 2000). The results indicated that topographically specific and sustained (30–60 min) increases in excitability of the motor system were possible through noninvasive PAS in humans by carefully timing cortical stimulation with somatosensory signals afferently propagated toward the cortex. PAS utility was subsequently reproduced in the spinal circuits (Taylor and Martin, 2009) by timing peripheral nerve stimulation so that antidromic potentials in motoneurons reached the cell bodies in the spinal cord shortly after the arrival of TMS-induced corticospinal volleys. Since that time, several studies have attempted to validate this phenomenon with mixed success and have studied PAS to drive plasticity in the neural circuits controlling upper and lower limbs of humans (Carson and Kennedy, 2013; Suppa et al., 2017).

PAS has demonstrated potential as a therapeutic intervention to strengthen residual circuits after spinal cord injury and promote functional recovery (Bunday and Perez, 2012; Urbin et al., 2017; Bunday et al., 2018). Studies have also used modified PAS protocols with mixed success in improving functional recovery after neurovascular insult, both in animals (Shin et al., 2008) and in humans (Castel-Lacanal et al., 2007, 2009; Rogers et al., 2011; Cho et al., 2016; Ferris et al., 2018; Palmer et al., 2018; Tarri et al., 2018). PAS initially showed great promise for rehabilitation; however, enthusiasm for this approach has been tempered by a lack of experimental rigor and inconsistent results (Alder et al., 2019). PAS has been shown to have a very high intersubject variability (Sale et al., 2007; McGie et al., 2014; Tarri et al., 2018), its effects are strongly dependent on mindful, persistent attention on the target limb (Stefan et al., 2004) or even failed to induce any consistent plastic effects (McGie et al., 2014).

### PAS in animals

Animal models are being developed to obtain a more robust and systematic evaluation of PAS effectiveness and underlying mechanisms. A few studies in rats have thus far shown that PAS could drive changes in corticomotor excitability toward both forelimb and hindlimb muscles (Shin et al., 2008; Mishra et al., 2017; Zhang et al., 2018). However, most studies have been performed under anesthesia, which can itself modulate plasticity (Yang et al., 2011; Huang and Yang, 2015), or using noninvasive methods in restrained animals. An animal model with chronically implanted electrodes allowing for a systematic study of the effectiveness of PAS in freely moving subjects did not exist thus far.

**Figure 2.**
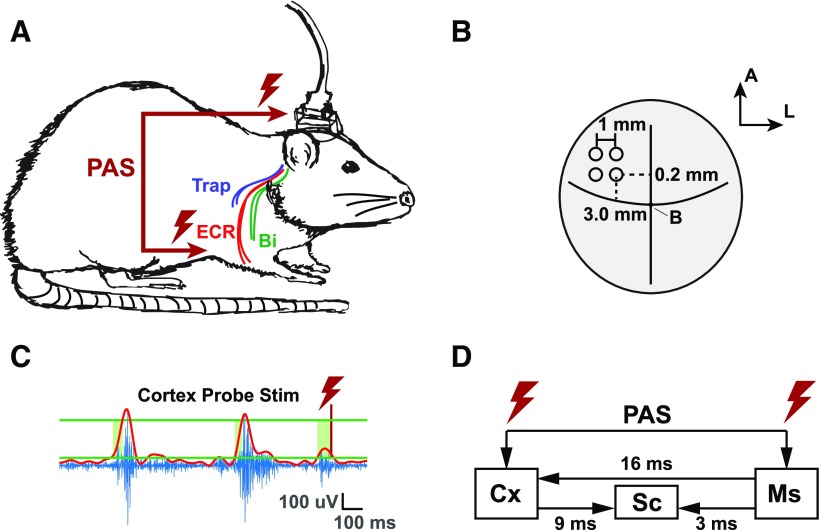
Experimental showcase. ***A***, Rats were chronically implanted with three pairs of subcutaneous stainless steel microwires to stimulate and record from the ECR, trapezius (Trap), and biceps (Bi) muscles contralateral to the cortical array. ***B***, Dorsal view of the rat brain, showing where we inserted the 2 × 2 platinum-iridium electrode array in the CFA of M1. Coordinates are anterior (A) and lateral (L) relative to bregma (B). ***C***, Corticomotor excitability was assessed before and after PAS using closed-loop, EMG-controlled motor cortical stimulation. The top envelope (red) of the EMG signal was calculated in pseudo-real time on the computer controlling data acquisition and stimulation using the MATLAB envelope function. Cortical stimulation was invoked when the envelope rose within 2–12 SDs above the mean signal (green horizontal lines) for at least 50 ms. The minimum time between stimulations was 1 s. ***D***, We know from our and previous studies under anesthesia that it takes ∼9 and 3 ms for signals issued from cortical and peripheral stimulation to arrive at the spinal cord, respectively. Peripheral stimulation of afferent fibers results in a volley of motor cortical activity after 16 ms. The presynaptic and postsynaptic activity offset at the levels of spinal cord and motor cortex for different interstimulus intervals were calculated based on these conduction latencies.

We aimed at developing such a model to perform a robust evaluation of PAS effectiveness in a context where stimulation is applied during naturally ongoing neuronal activity. We applied PAS in freely behaving rats with chronically implanted cortical and intramuscular electrodes (Fig. 2*A*). By holding other parameters constant (stimulation amplitude, frequency, number of pulses), we tested a wide range of interstimulus intervals, hypothesizing that certain timings would result in corticomotor potentiation, but failed to significantly modulate corticomotor excitability as predicted by the STDP model.

## Materials and Methods

### Animals and surgical preparation

All animal procedures were performed in accordance with the regulations of the Université Laval animal care committee. Nine Long–Evans rats and one Sprague Dawley rat (all male) were housed under 12 h inverse daylight cycle with food and water available *ad libitum*. Animals were individually housed to prevent implant damage. One-hundred fifty experimental sessions were planned in 10 animals during the dark (active) phase of their daylight cycle (coinciding with our work day) to investigate the effectiveness of 15 interstimulus intervals (ISIs), including control stimulation protocols. However, due to rare implant failure, some ISIs were not tested in all the rats. All ISI conditions were tested in a minimum of five animals, and an average of seven to eight (Extended Data Fig. 4-1). Our PAS intervention typically targeted the extensor carpi radialis (ECR) muscle. However, in some animals, we used pairs of EMG wires implanted in more proximal locations (biceps or trapezius muscles). The distribution of muscles tested within the full dataset is shown in Extended Data Figure 4-2.

### Chronic PAS implantation surgery

During aseptic surgeries performed under isoflurane anesthesia, rats were implanted with 1 × 1 mm custom square arrays of four 80/20 platinum-iridium electrodes, each 75 μm in diameter and an approximate impedance of 20 kΩ. The electrodes were inserted 1.5 mm deep into the caudal forelimb area (CFA) of the primary motor cortex (M1) by a stereotaxic craniectomy, centered at 0.7 mm anterior and 3.5 mm lateral relative to bregma, and the surrounding exposed dura matter was covered with silicone gel for protection (Fig. 2*B*). This allowed us to perform intracortical stimulation using an isolated constant current stimulator (model 2100, A-M Systems). Three pairs of PFA-coated multistranded stainless steel wire electrodes (A-M Systems) were inserted into the contralateral ECR, biceps brachii, and trapezius muscles (the latter two serving as alternative muscles in case the ECR electrode failed). EMG and cortical electrodes were presoldered to either an InVivo1 MS12P or a SAMTEC 2 × 7 connector, which were secured to the skull with dental cement and six bone screws as anchors. A posterior skull screw served as the ground electrode for cortical monopolar stimulation. An additional reference electrode for the EMG measurement was embedded subcutaneously in the upper back. Animals recovered undisturbed for a week after implantation prior to testing and were given time to familiarize themselves with being connected. EMG electrodes were then tested for recording quality, and two electrodes in each cortical array with the lowest stimulation intensity required for motor evoked potentials (MEPs) in the target muscle were determined prior to data collection (barring electrode failure, the same two were used in a bipolar configuration for all of the experiments for that rat).

### Study design and experimental paradigm

We used a repeated-measures randomized block design (same rat tested on all ISI conditions in a randomized order) to test the effect of STDP timing condition on the change in integral of the averaged MEP response after the PAS experiment.

We tested nine rats with chronic implants (the implant for one rat failed prior to data collection). Each rat was to be tested once in each condition. To account for possible order effects inherent to a within-subjects design, the order of testing conditions was randomly assigned using the *randperm* function in MATLAB (Extended Data Fig. 4-1). One condition (ISI, −15 ms) was added at the end for six rats that had all data collection completed, based on another study (Zhang et al., 2018) that showed a promising timing condition and was published while data collection was in progress. For rats with which data collection had not started yet, a rerandomization was performed to integrate this new condition. For each rat, each test was separated by ∼24 h to minimize carryover effects between previous paired stimulation interventions. *A posteriori* analyses verified that there was no cumulative effect of PAS (Extended Data Fig. 4-4).

We tested the following four control conditions: three PAS controls, involving (1) cortical stimulation only, (2) peripheral stimulation only, and (3) no stimulation, as well as (4) one extralong ISI timing control involving paired stimulation of the motor cortex and the contralateral peripheral muscle offset by +505 ms. We reasoned that if timing during paired stimulation was the driving factor behind plasticity, and not the pairing of stimulation per se, this condition should have a null effect comparable to the previous control conditions.

**Figure 3.**
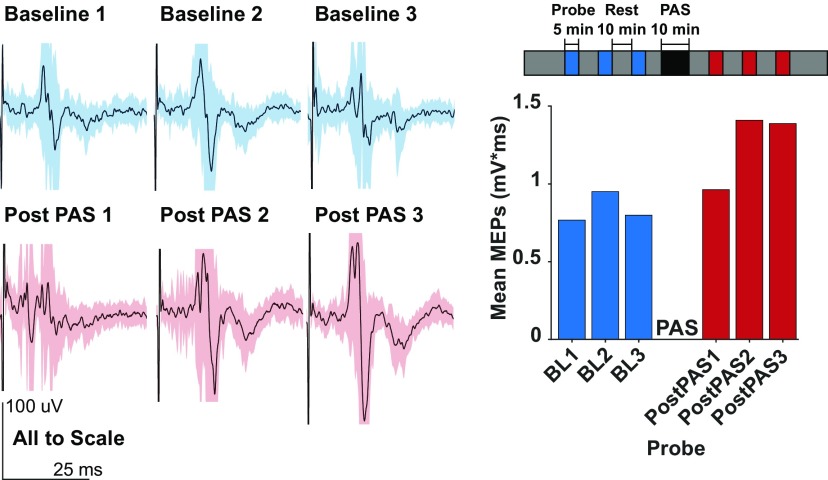
Example experiment-level result: MEPs recorded in the right ECR of one rat, obtained from electrical cortical stimulation for each probe. Shaded areas in light blue and red indicate 1 SD about the mean. Inset, Each session began with three 5 min probes in which we performed closed-loop EMG-dependent cortical stimulation to assess baseline MEP amplitudes, each separated by 10 min. The PAS session itself, involving 300 pairs of stimuli to the cortex and the muscle at a rate of 0.5 Hz, took ∼10 min. This was followed by three post-PAS probes so we could assess corticospinal excitability up to 30 min after paired stimulation for each interstimulus interval. After each experiment, we manually verified all MEPs using custom software and excluded traces with movement artifacts or noisy EMG signals.

Each experiment followed a fixed schedule (Fig. 3, inset). After connecting the rat to the hardware interface, we completed three 5 min “probes” to assess the corticomotor excitability prior to the PAS intervention (see following section). The probe was completed when 30 stimulations were delivered or 5 min had elapsed, whichever came first. Probes were separated by 10 min each. After each PAS intervention, three post-PAS probes were completed in the same manner to assess the excitability of the corticomotor system after paired stimulation. We allotted 2 min for wire switching and software changes, immediately before and after each PAS intervention.

### Probe assessment of corticomotor excitability

To assess corticomotor excitability before and after PAS, we compared the size of MEPs obtained from cortical stimulation using a closed-loop stimulation protocol (Fig. 2*C*). As demonstrated by Darling et al. (2006), cortical stimulation during a low level of muscle contraction (5% or 10% of their maximal voluntary contraction) reduces MEP variability, compared with fully relaxed conditions. Drawing inspiration from this, we designed a protocol to stimulate during low levels of muscle contraction in the target muscle. To this end, the EMG activity in the target muscle was continuously measured, and cortical stimulation was triggered in real time if the activity reached within 2–12 SDs above the baseline (defined as the mean value of the rectified EMG signal measured over 2 s when the limb was fully relaxed, during sleep, or during sustained rest behavior with no weight bearing in each rat), and if the EMG activity was on the rising phase (i.e., contraction was being initiated during free behavior as opposed to when the muscle was relaxing from a previously larger contraction). The baseline calibration was performed on each rat prior to data collection and recalculated as necessary if we suspected a change in baseline noise. This allowed us to customize the excitability assessment to each rat to adjust for slight differences in electrode placement or impedance across days. However, the EMG assessment window was never changed within a PAS experiment. The 2–12 SD range above baseline effectively restricted the conditions for stimulation within a low to moderate level of voluntary activation of the corticospinal system achieved during free behavior (walking/grooming/exploring). This approach provides the means to stimulate under consistent conditions of corticospinal activity, in an animal model where behavioral instructions cannot be clearly provided such as in human studies. To do this, we used the envelope function in MATLAB, which calculated the peak envelope of the filtered data with a moving spline over the downsampled local maxima of the previous 32 data points. Cortical stimulation was contingent on the EMG envelope crossing the predetermined activity threshold. The variability was still high albeit reduced after applying the closed-loop stimulation protocol, so we averaged all three baseline measurements during the statistical analysis to obtain an overall assessment of corticomotor excitability prior to the PAS intervention. We recorded from different muscles, depending on the location where we obtained the best quality MEPs (Extended Data Fig. 4-2). We always used the same muscle involved in the PAS intervention to provide the closed-loop control for the corticomotor excitability assessments. When available, we chose the ECR as the PAS target muscle, but fell back on the biceps or trapezius, respectively, if electrode malfunctions or failure to evoke MEPs in the more distal muscles prevented their use. In one rat, we used a monopolar EMG recording configuration resulting in an EMG signal contaminated with cross talk from cardiac activity. We manually adjusted the upper and lower limits of the EMG window, enabling probe stimulation in a manner that better reflected a low-amplitude muscle contraction.

### Electrophysiological data acquisition and stimulation configuration

Independent paired electrical stimulation protocols were achieved through two A-M Systems 2100 stimulators, each connected to separate pins on an InVivo1 commutator through a custom-made breakout board interface. Multichannel recording was made possible by routing the EMG signal into a Brownlee Precision Model 440 Instrumentation Amplifier. Within this unit, a signal gain of 100, a bandpass filter between 50 Hz and 1.0 kHz was used for EMG, and bandpass of 1–300 Hz for local field potential (LFP) signals. A 60 Hz notch filter was applied. This output signal was split in two, with one copy being routed into a Powerlab 8/sp unit by AD Instruments and further processed with a 10 Hz high-pass filter before being saved. The second copy was routed into a National Instruments Digital to Analog Converter (DAC) SCB-68A system, which was operated via custom MATLAB software. We used the DAC system and MATLAB software to initiate all probe and PAS stimulation protocols via the trigger input ports on the A-M Systems stimulators.

### Latency measurements and sign convention for spike-timing experiments

To confirm the conduction latencies, we completed a series of acute experiments under anesthesia, in rats with a similar weight and size to those used for chronic implants. First, to measure the antidromic conduction time in motoneurons between the muscle and the spinal cord, we performed an acute experiment under urethane anesthesia to record and stimulate between the spinal cord and the ECR muscle, respectively. We exposed the dorsal spinal cord between the C4 and C6 regions by performing a laminectomy and deafferented the C3 to C7 segments by cutting the dorsal roots to isolate antidromic propagation instead of conduction along afferent sensory fibers. With the dura intact, we inserted a tungsten electrode, 127 μm in diameter, into the C5 region ipsilateral to the right forelimb, 1.0 mm lateral to the midline. We also inserted a pair of EMG electrodes in the right extensor carpi radialis using the same method as in the chronic implants. Stimulation of the spinal cord C5 region using single pulses led to an isolated wrist extension in the forelimb of the rat, verifying the location of the ECR motoneuron pool for efferent connections (Tosolini and Morris, 2012). Following this, we stimulated the EMG electrodes and recorded LFPs from the electrode site in C5. Filtering parameters for the LFP recording included a bandpass of 1–300 Hz, with a 60 Hz notch filter and a gain of 100. Data were averaged across 200 stimulations. We determined the antidromic motoneuronal propagation delay to be 3 ms (Extended Data Fig. 2-1*A*).

10.1523/ENEURO.0396-19.2020.f2-1 Figure 2-1 Spinal and cortical evoked potentials from peripheral stimulation. ***A***, The latency of the deepest trough response at 3 ms in the C5 region of the spinal cord, averaged across 200 stimulations. Note that a DC offset in the baseline signal was manually adjusted here. ***B***, Measuring the latency of the deepest trough response at 16 ms in the cortex, averaged across 360 stimulations. Download Figure 2-1, EPS file.

With an average MEP latency of 12 ms for ECR deriving from cortical stimulation of the intact animal, and a 3 ms peripheral efferent conduction time, we estimated a latency of 9 ms for a cortical stimulation-induced descending volley to reach motoneurons, including synaptic integration time.

In a second acute experiment with a different animal under ketamine/xylazine anesthesia, we measured the time a neuronal volley requires to reach the cortex after muscle stimulation. In an intact animal, we recorded LFP responses in M1 following intramuscular stimulation (Extended Data Fig. 2-1*B*). We postulated that the peak of the initial negative inflection in the local field potential from contralateral muscle stimulation reflected the time at which the greatest neural activity is observed among the postsynaptic neurons in the cortex. Again, we inserted a pair of EMG electrodes into the right extensor carpi radialis, then inserted one platinum-iridium electrode 1.5 mm dorsoventrally into M1, centered at the array coordinates of the rats involved in the PAS experiments. A reference electrode ∼1 mm lateral from the first was positioned on the surface of the dura. Both cortical electrodes were connected by a common ground at the skull screw, and LFPs were measured by calculating the voltage differential between the cortical electrodes with the same LFP recording parameters described above. Stimulation was delivered to the EMG electrode in the right ECR through bipolar single pulses with a 0.2 ms duration, repeated at 0.5 Hz. Data were averaged across 360 stimulations. The afferent latency from ECR stimulation to the peak of the cortical evoked potential was 16 ms (Extended Data Fig. 2-1*B*).

Using these conduction latencies, we chose a set of stimulus intervals that would result in various presynaptic and postsynaptic timings relevant to the rules of spike timing-dependent plasticity, either at the cortical and/or spinal levels. The full list of ISI conditions tested can be found in Extended Data Figure 4-1. Our experimental design and results followed the convention that a positive latency means the periphery was stimulated after the cortex by that time difference. These stimulation offsets lead to physiological offsets calculated at the levels of the spinal cord and cortex; positive latencies result in presynaptic activity that preceded postsynaptic activity at the specified location.

**Figure 4.**
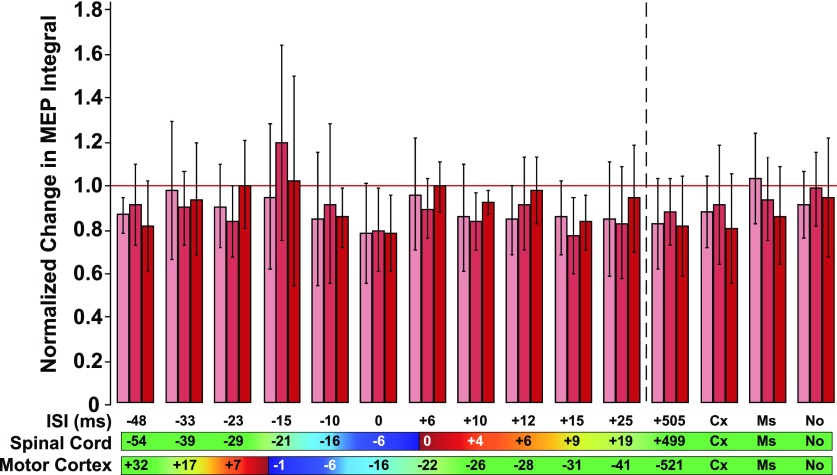
PAS does not significantly potentiate MEP responses *in vivo*. Grouped bar plot, depicting that for Post 1 (2 min after PAS, light red), Post 2 (17 min after PAS, medium red), and Post 3 (32 min after PAS, dark red) sessions, there were no significant differences between STDP experimental conditions and control conditions. Error bars are 95% confidence intervals about the mean. The horizontal reference line marked in red signifies no change between that post condition and the baseline average. Control conditions are shown to the right of the vertical dotted black line, to separate them from the ISI conditions tested to the left (Cx, cortical stimulation only; Ms, muscle stimulation only; No, no stimulation). ISI refers to the latency between stimulation of the cortex and the muscle. Here, positive numbers refer to muscle stimulation occurring after cortical stimulation. “Spinal Cord” numbers are the estimated latencies between the arrival of the descending volley onto the motoneurons in the spinal cord and the arrival of the antidromic action potentials evoked from muscle stimulation (positive if orthodromic arrives before antidromic); “Motor Cortex” numbers are the estimated latencies between the arrival in M1 of peripheral stimulation-induced afferent activity and the motor cortex stimulation (positive if peripheral afferent signal arrives before cortex stimulation signal). The colors on the horizontal bars at the bottom indicate conditions expected to induce LTP-like effects (red), LTD-like effects (blue), or no significant modulation (green) based on the Hebbian STDP model.

10.1523/ENEURO.0396-19.2020.f4-1 Figure 4-1 Study design for chronic experiments and *a priori* randomization order (number within each cell). The final sample sizes for each PAS condition are written to the left. Sessions used in the final dataset are shaded in gray. Download Figure 4-1, DOC file.

10.1523/ENEURO.0396-19.2020.f4-2 Figure 4-2 Muscle distribution. Distribution of muscles used as PAS target (ISI Conditions, Latencies are Stimulation Offsets). *PAS muscle stimulation component was between one EMG electrode and reference. Download Figure 4-2, DOC file.

### PAS intervention

We used a PAS protocol of 300 paired stimulations to the motor cortex and designated peripheral muscle, using single pulses of biphasic electrical stimulation 0.2 ms in duration, separated by 0.5 Hz. We note that this is on the higher end in terms of the number of paired stimulations compared with previous protocols, and is delivered at a higher frequency, but we reasoned, in the absence of evidence otherwise, that any effect that may be present due to paired stimulation should be enhanced using this slightly more intensive protocol.

Cortical stimulation intensity was set at 1.25 times the threshold for an MEP and muscle stimulation at 1.5 times the threshold to elicit a visible twitch (the mean motor threshold across all experiments was 790 μA for cortical stimulation and 1.8 mA for muscle stimulation). Thresholds were operationally defined as the minimal stimulation intensity required to induce a response >50% of the time. All PAS experiments were completed in the home cages of the animals with a modified cover that enabled us to pass the tethering cable during free behavior (consisting mostly of walking, grooming, and exploring, and sometimes sleeping).

### MEP measurement

Raw EMG data were saved and processed offline in LabChart Version 7, and custom scripts were written in MATLAB. We plotted all individual responses for each cortical stimulation and manually excluded trials for which there was significant excessive movement artifact and/or lack of EMG signal (this was rare, and the most likely reason was due to an intermittent connection with a faulty cable, which was repaired or replaced promptly). The resulting set of verified MEPs for each probe was collected for further analysis.

MEP amplitudes were initially quantified with the following three different methods: (1) the peak-to-peak value of individual EMG responses (the literature standard); (2) the mean value of the integral of individual rectified EMG responses, measured over a tailored time window following stimulation; and (3) the integral of the averaged rectified EMG responses over the same time window. Every individual response to cortical stimulation was first manually screened to exclude any EMG traces containing large movement artifacts or other obvious contamination. In pilot analyses from our earlier experiments (data not shown), we assessed qualitatively that the calculation method did not much impact the normalized changes in the MEPs, so we proceeded with taking the integral of the average response for the probe (method 3 above). We reasoned that this approach was most effective in capturing both unimodal and multimodal MEP responses. This decision was made prior to the pooled study data analysis. In summary, the MEP values reported here were thus calculated by first rectifying the filtered EMG signal, then averaging the activity from all stimuli within a probe postscreening, and then calculating the integral of the resulting signal (Fig. 3). The software described in this article is freely available online at https://github.com/ethierlab (Windows 10). The code is available as Extended Data, if required.

### Statistical analysis

Statistical analysis and visualization were completed using SAS Software, version 9.4 for Windows, Minitab 18 for Windows, and R 3.6.1/RStudio 1.2.5019 for Windows. We completed a mixed-design ANOVA with repeated measures on the normalized data to test the main effects of ISI timing condition (CONDITION) with 15 levels (1 level for each of 11 timings and four control conditions) as well as PAS probe (SESSION) with three levels (2, 17, and 32 min after PAS). We also tested for any interaction effects between CONDITION and SESSION. A random effect on the rat was used to account for the randomized block design. The level of significance for the mixed ANOVA was fixed at *p* < 0.05. Type III fixed effects are reported in Table 1, obtained through the Restricted Maximum Likelihood (REML) estimation method. Data from one rat in the ISI +6 condition was removed from the statistical analysis because of poor data quality (very few MEPs in each probe). Normality was assessed on standardized residuals using graphical methods.

**Table 1:** Summary of statistical analyses

Figure	Type of test	Term	Data structure	df Numerator	df Denominator	*F* Value	*p* Value
4	Mixed-effects ANOVA	a. Session × Condition	Model residuals normal	28.00	250.92	0.53	0.976
		b. Condition		14.00	253.60	1.56	0.092
		c. Session		2.00	250.92	0.08	0.921

Mixed-effects model analysis, fixed effects (type III) using the REML method. df = Degrees of Freedom.

## Results

### Failure of spike timing to modulate cortical and spinal plasticity

We tested a wide range of STDP-relevant intervals between cortical and peripheral stimuli (ISI conditions) in a randomized fashion for each rat and used an EMG-controlled closed-loop method to measure preintervention and postintervention MEPs. We found no significant modulation of corticospinal excitability using our PAS intervention *in vivo*. We analyzed the MEP amplitudes obtained from cortical stimulation probes before and after each PAS intervention using a mixed-model ANOVA on the normalized data. Probe time (SESSION) was considered a repeated-measures fixed factor, and ISI condition (CONDITION) was the second fixed factor. We included a SESSION × CONDITION interaction term in the statistical model, and a random factor for the rat accounting for the randomized design. We did not find a significant effect of our PAS intervention for any of the timings we tested (Fig. 4). Statistically, there was no significant interaction (Table 1, a) between SESSION (preintervention and postintervention MEPs) and CONDITION (*F*_(28,251)_ = 0.53, *p* = 0.98)), meaning that there was no ISI condition for which the intervention resulted in a statistically significant change in corticospinal excitability over assessment time. There was no main effect of ISI CONDITION (Table 1, b; *F*_(14,254)_ = 1.56, *p* = 0.09) independent of the time at which the MEP was measured postintervention, indicating that the ISI condition did not have a significant effect on the MEP amplitude. There was no significant main effect of SESSION (Table 1, c; *F*_(2,251)_ = 0.08, *p* = 0.921). Qualitatively, our PAS protocol did not induce changes in MEP size consistent with STDP in individual rats (Extended Data Fig. 4-3). In summary, our statistical analyses did not support the efficacy of PAS under these conditions.

10.1523/ENEURO.0396-19.2020.f4-3 Figure 4-3 No individualized effect of PAS. Analogous plot to Figure 4, but paneled by rat, demonstrating that there was no effect consistent with STDP from our PAS intervention even on the level of individual animals. Download Figure 4-3, EPS file.

10.1523/ENEURO.0396-19.2020.f4-4 Figure 4-4 No cumulative effect of PAS. Baseline MEP amplitude on days immediately following a PAS session was not correlated to MEP changes in that session. The “Within-Session MEP Ratio” is defined as the normalized change in averaged corticomotor excitability across a given PAS intervention WITHIN an experimental day (N), and the “Next-Day Baseline MEP Ratio” is defined in the same way but BETWEEN the baseline average on day N and the baseline average on day N + 1 (consecutive calendar day). While processing the data BETWEEN days, we ensured that there were no changes in cortex stimulation intensity, muscle stimulation intensity, the electrode leads used for both sites, the type of EMG recording (monopolar or bipolar), and the EMG range for closed-loop stimulation. If any of these parameters changed between days, those data points were excluded. This ensured full homogeneity in the stimulation conditions used to calculate the appropriate quotients. All of the above parameters were held constant by design within a particular experimental day. By testing the correlation between these two ratios, we could directly assess whether the change induced by any particular PAS protocol on a given day is related to the change in baseline excitability across days. The two variables were not strongly correlated (*r* = 0.18) and the relationship was not significant (*p* = 0.21), even after winsorization to remove outliers (*p* = 0.19), confirming there was no carryover effect of our PAS intervention. Download Figure 4-4, EPS file.

### Control experiments

Four different control protocols where we did cortical/muscle stimulation in isolation, no stimulation, and maintained a large offset between paired stimuli, respectively, did not significantly alter corticomotor excitability (Fig. 4, conditions to the right of vertical dotted line). Interestingly, we noted a trend toward a depressive effect for the cortical stimulation only (mean ratio post-stimulation/pre-stimulation, 0.87) and ISI +505 ms stimulation (0.84) conditions, but less so for the muscle stimulation only (0.94) and the no-stimulation conditions (0.95).

## Discussion

There exists a mixed literature on human PAS and several variations of the original protocol (Stefan et al., 2000), with some convincing reports demonstrating its effectiveness for inducing at least transient changes in corticomotor excitability (Taylor and Martin, 2009; Bunday et al., 2018), and others showing ineffective interventions or highly subject-dependent results (Müller-Dahlhaus et al., 2008; McGie et al., 2014). Our own results support the latter findings. Our PAS protocol, with parameters inspired by typical interventions in humans, was ineffective at modulating plastic changes in corticospinal excitability. There was no significant interaction between fixed factors, leading us to conclude that our PAS protocol was ineffective overall in potentiating corticospinal connections.

### PAS parameter space

Setting aside spike timing, the entire parameter space for a PAS intervention protocol is vast, with no known physiological principles guiding a specific combination of stimulation intensity and frequency and/or number of repetitions over another. Consistent with most studies, we chose above-threshold but submaximal stimulation amplitudes (1.5× and 1.25× motor threshold for muscles and cortex, respectively). We decided on a PAS protocol with a number of paired stimulations (300) and stimulation frequency (0.5 Hz) on the higher end compared with most other published protocols (Suppa et al., 2017). We reasoned that, if anything, this would enhance any PAS effects. It would be possible but counterintuitive that these differences reduced the likelihood of inducing plastic changes.

### Overall depressive trend

We observed that MEPs after PAS interventions were generally smaller than the average of the baseline measurements. This trend was also present for control conditions involving cortical stimulation alone, but less so when the rats received no stimulation or muscle stimulation only in place of PAS. These observations can be apprised given the evidence that single pulses of peripheral electrical stimulation are insufficient to change corticomotor excitability with or without coincident voluntary contraction in humans (Saito et al., 2014), and that higher frequencies are needed for supraspinal effects (Grosprêtre et al., 2017). These results indicate that the probes themselves had no effects, but that all stimulation interventions involving cortical stimulation induced a trend toward an LTD-like effect. The effect was not statistically significant, but it would be consistent with depression of motor cortical excitability observed after low-frequency (1 Hz) TMS in humans (Chen et al., 1997). A small decrease in cortical excitability would have reduced any LTP-like effects and enhanced LTD-like effects induced by the PAS protocol as predicted by STDP. In other words, a general dampening cortical effect induced by the slow repetition of our stimulus pairs could prevent us from detecting any LTP-like effect but would presumably make PAS-induced LTD-like effects even more prominent. As our statistical analysis did not reveal any significant changes in MEP sizes in either direction, we conclude that our PAS intervention did not induce plastic changes following STDP rules.

### Closed-loop assessment

Another plausible explanation for our negative results is the high intrinsic variability observed in the MEP responses of our rats during free behavior. Our EMG-based closed-loop preintervention and postintervention assessment probes were specifically designed to assess the excitability of the corticomotor system at relatively similar, low levels of EMG activity (∼5–15% of maximum EMG amplitude observed under free behavior). The aim was to minimize MEP variability by avoiding stimulation in different conditions of corticomotor excitability, such as during a strong voluntary contraction or during reciprocal inhibition acting on the recorded muscle. However, the PAS intervention itself was not completed in an EMG-dependent manner, because we could not record and stimulate muscles simultaneously with our setup. Perhaps also applying the closed-loop approach to the paired stimulation would have allowed for a more systematic and reproducible recruitment of neuronal elements, thereby leading to more reliable PAS effects.

### Stimulation models and specificity

In our chronic PAS model, we inserted electrodes directly into the target muscle and validated this approach in an acute experiment to verify that electrical stimulation of the muscle fiber was sufficient to generate antidromic volleys backpropagating to the deafferented spinal cord. Compared with direct nerve stimulation, intramuscular stimulation may result in a small difference in the relative timing of stimulation-induced antidromic motoneuron activation and orthodromic afferent activity. In addition, direct nerve stimulation can recruit a larger number of fibers of all modalities, not limited to a specific target muscle, but including all motor and sensory fibers traveling in the nerve at the chosen stimulus location. These differences in peripheral fiber recruitment may have contributed to the apparent inconsistency between our results and those of others showing the effectiveness of PAS using electrical stimulation in rodents (Mishra et al., 2017).

With respect to the PAS literature, we can hypothesize there may be intrinsic differences in MEP variability between noninvasive stimulation (TMS, the standard technique for PAS in humans) and invasive stimulation [intracortical microstimulation (ICMS)] methods due to different circuits being recruited. ICMS, although having a greater spatial and temporal resolution than noninvasive methods of neural activation such as TMS, is also nonspecific in the sense that it activates all types of neurons and other cell types. The exact recruitment patterns of the cortical circuits are of very little theoretical importance for PAS interventions targeting spinal circuits, as long as a corticospinal volley occurs in a timely manner relative to peripheral stimulation. Therefore, especially since it was previously used successfully in rats (Mishra et al., 2017), it would be surprising if our use of ICMS was a factor in explaining our negative results.

Some electrophysiology studies have suggested that there are both monosynaptic and polysynaptic connections onto the corticospinal motoneurons of rats (Elger et al., 1977; Liang et al., 1991; Hori et al., 2002), but more recent work has suggested that the rat corticospinal tract is exclusively polysynaptic (Alstermark et al., 2004). Although this is a major physiological difference between rats and primates, we believe that this is not a critical factor to explain differences between our negative results and successful human PAS. In addition to the variable conduction time between individual fibers, a polysynaptic pathway will increase the temporal spread of action potentials in a stimulation-induced volley. Furthermore, the ascending afferent sensory pathway in humans is polysynaptic (Abraira and Ginty, 2013), and yet PAS is still effective when TMS is timed with the arrival of afferent volleys in the cortex (Stefan et al., 2000). By the same token, we expected that a polysynaptic descending pathway would not prevent us from timing the descending volley with antidromic motoneuron activation.

### Opposing plastic changes along the corticomotor pathway

Thinking along these lines, however, the ISI timing offset of the paired stimulation dictates the target location of plasticity. In an ideal world, the effects will be localized only to one target area. However, since the corticomotor contains multiple synaptic connections, any given ISI condition predicted to induce LTP-like changes at one site according to Hebbian STDP (e.g., the motor cortex) could lead to LTD-like effects at the second site (e.g., the spinal cord for instance) and vice versa. In rats, we estimated the interval between PAS-induced presynaptic and postsynaptic activity at the cortex and the spinal cord for given cortical and peripheral stimulation intervals. These opposing effects are reflected in the lack of situations where LTP-like effects (Fig. 4, numbers shaded in red regions) can be predicted at both spinal and cortical levels. This competition between potentiation and depression at different locations may reduce the PAS effectiveness to induce a net increase in corticospinal excitability. Because of the noninvasive nature of human PAS experiments, this can potentially be an explanatory factor for the variance in PAS effectiveness observed in the clinical data. This issue can be dissected in animal models with terminal experiments, but addressing this issue *in vivo* will require advances in our stimulation methods to be simultaneously noninvasive, yet highly spatially specific. The goal here would be to isolate the bookends of the paired stimulation just bounding the targeted synapses. That would be a seminal advance in addressing the utility of PAS *in vivo*.

### Seeking the perfect storm

Voluntary effort itself has been shown to be a necessary driver for potentiation in humans for specific PAS protocols (Kujirai et al., 2006), with two proposed mechanisms being the reduction of intracortical inhibition networks coincident with contraction or the facilitatory effect of attention via the activation of memory systems (Stefan et al., 2004), but this is contradictory to earlier cited findings that PAS works well under anesthesia in animals (despite the differences among species). The effect of known neuromodulators on PAS, such as dopamine, should not be underestimated, particularly because of its direct role in mediating neuronal potentiation (Yagishita et al., 2014) and its broader implications in maintaining attention (Suppa et al., 2017). Additional factors influencing PAS effectiveness are numerous, and may include even the time of day—a study in humans showed that PAS sessions performed in the afternoon were significantly potentiated in one study, whereas sessions completed in the morning did not (Sale et al., 2007). In that article, variance was attributed to circadian effects and specifically the inhibitory effect of cortisol on plasticity. These examples drive home the point that our knowledge of what coincident factors are required to induce LTP-like potentiation remains limited, and, based on our study, future studies should likely not be restricted to simple application of Hebbian principles; it may not be enough.

PAS has also been reported to exhibit high variance depending on the subject being tested. McGie et al. (2014) conducted a noninvasive PAS study in humans, with the goal of comparing different paired stimulation protocol frequencies (McGie et al., 2014). Tarri et al. (2018) studied the effect of PAS in humans as a therapeutic adjunct to stroke using a randomized double-blind controlled approach (the CIPASS Trial; Tarri et al., 2018). Both groups reported high between-subject variability in PAS outcomes but found no consistent effect of PAS targeting spinal circuits, attributing the variability observed to individual factors such as the lesion size/location, and different rehabilitation intensiveness, both influencing the physiological capacity available for PAS effects. Importantly, the degree of muscle facilitation can vary greatly even within the same participants across repeated PAS sessions (Tarri et al., 2018). These studies emphasize the mercurial nature of PAS effectiveness even within individuals and the highly stereotyped/specialized conditions necessary for consistent beneficial effects to become apparent. It may turn out that a conjunction of multiple concurrently acting factors is necessary to facilitate PAS potentiation under free behavior in animals.

### Conclusion

In conclusion, our data do not support the effectiveness of PAS in promoting plasticity through the Hebbian STDP model in freely behaving rodents. Our initial goal was to develop a clinically relevant animal model for paired stimulation that would have allowed more detailed studies and optimize interventions. Although the model itself was developed successfully, this series of experiments suggested that an open-loop PAS intervention in a freely moving animal is not effective to reliably drive plasticity in the corticospinal system. Our results highlight the complexity of associative plasticity and demonstrate that forced coincidence of neuronal activity is not sufficient to reliably potentiate corticospinal excitability. Future research will need to investigate whether other variations in the PAS parameter space, reduction of interference from ongoing neuronal activity, or manipulations of neuromodulators may be required to drive corticospinal potentiation more reliably. This will determine whether PAS indeed has potential as an interventional measure for modulating corticomotor plasticity.

## Synthesis

Reviewing Editor: Trevor Drew, University of Montreal Decisions are customarily a result of the Reviewing Editor and the peer reviewers coming together and discussing their recommendations until a consensus is reached. When revisions are invited, a fact-based synthesis statement explaining their decision and outlining what is needed to prepare a revision will be listed below. The following reviewer(s) agreed to reveal their identity: Numa Dancause.

Reviewer #!

This study investigated the impact of PAS in awake, uninjured rats. Several interstimulation intervals between the cortical and muscle stimulations were systematically applied in protocols that included 300 paired stimulations using suprathreshold intensities at the cortex and muscle. The effect of the PAS was calculate with probes taking into consideration the target muscle activity, before and after the stimulation. The authors found that PAS did not induce significant effects at the group level. The manuscript is generally well written and includes a good introduction and fair discussion. The study is methodologically sound and so the results are convincing and important, although they will not be ‘crowd pleasing’. I have a few questions and suggestions at this point.

Major

I have two major comments. Both are related to the lack of analyses of individual animals' data.

First, it is assumed that the delay between protocol was sufficient to prevent cumulative effects (P6Ln118). This could be verified and substantiated by the data. Were data inspected over time for each rat to verify that there were no trend with the cumulation of protocols?

Second, there is no group effect for the protocol but, there could be individual differences, much like there seems to be in humans. It would be good to confirm that PAS was ineffective in all rats. One alternative possibility is that some protocols were effective in some rats and other protocols in other rats. If this is the case, perhaps there is a strong message for individualisation of protocols based on specific responses could be made.

Minor:

P2Ln41. Why is PAS considered non-invasive? Here the cortical stimulation is invasive to my understanding (also see below).

P2Ln45. ‘in man’ should be ‘in human’

P4Ln83. The previous sentence report n=9 and here n=10 (it goes back to n=10 on Ln112). I understand that the implant failed in one rat, but this should be specified here or the n should change from 10 to 9 when the failure is reported. In addition, ‘controls’ in this sentence should be better defined (e.g. ‘control stimulation protocols’?)

P5Ln94. Are these surface or intracortical electrodes? If the later, how deep are they. Also what is the impedance and was it constant current delivered? What is the estimated radius of the stimulation and how does it compare to the size of the forearm area in this rat strain? How do these numbers compare to what is done in humans?

P6Ln125. ‘previous PAS control conditions’ These control conditions are specifically not PAS.

P6Ln137. ‘within [2 12] SD above’ The reasoning for the selection of these metrics is not completely clear (why 12 instead of any other value?). Also it is unclear how the ‘fully relaxed’ state is defined and how this baseline is collected. Is this done on trial basis or once per session prior to collecting the probes or once in the rat's life time? Also how is this different or similar to human studies? Finally, cortical stimulation in rats typically induces EMG responses in multiple muscles. How was the muscle for the close loop probe selected? Was the level of activity in other muscles controlled for?

P7Ln138. ‘fully relaxed), and the if EMG activity’ probably switch the ‘if’ and ‘the’

P10Ln221. ‘Raw EMG data was saved’ ‘were’

Figure 1. It is not clear from the text if this figure previously published. In any case, it seems inconsistent with the description and it is actually quite confusing. The figure shows that with simultaneous stimulation (delta t=0) you have maximum LTP and LTD. However, maximal LTP is expected when the pre-synaptic activity repetitively occurs several ms prior to post-synaptic activity (as stated in the figure legend).

-

Reviewer #2

This study instrumented rats for chronic motor cortex and peripheral stimulation to determine if PAS was effective in augmenting MEP amplitude, according to a spike-timing dependent plasticity timing rule. Surprisingly, no response changes were observed over a wide range of intervals that should have covered the intervals shown in other studies to have the largest facilitation and suppression.

The study was performed with much care. The authors used a closed loop recording approach, whereby cortical stimulation only occurred when the EMG from the recorded muscle was within a certain window (2-12xSD of baseline). The chronic preparation appeared to be stable and enable examination in awake animals.

The authors argue that PAS efficacy is far from uniform across the literature, in both humans and animals. In the Discussion, the authors consider potential PAS dependencies. Several reasons for why no response facilitation was observed are described, from neuromuscular fatigue (most responses across all intervals and the post-PAS period were below baseline) to the nature of the closed loop protocol. I cannot add to that.

My major concern is that the experimental design was to have each rat experience all ISIs and to pool data across all animals. Whereas that is reasonable, individual animal ISI effects are not considered. Animals, and humans, vary in terms of being non-responders in many different sorts of activity-related and plasticity studies. For example, the efficacy of meta-plasticity of TMS in humans has been shown to be related to BDNF variants (e.g., Val55Met). And, as discussed in the Introduction and Discussion, this is a literature basis for variability in PAS. There may be timing efficacy variability across animals. When data are pooled, a weak (or even strong) effect in some animals, can wash out. I do not think the statistical analysis considered this. Moreover, the large number of intervals examined will further contribute to individual/group loss of statistical power. I would like to know if each of the subjects in the study had a flat MEP relationship with ISI. I get it that PAS in this model did not stand the authors' test of pooling data. However, demonstrating that there was no effect in each animal is, in my opinion, important to document.

Minor concerns.

Line 7 (and related parts of the text). It is indicated that PAS was assessed using closed loop. This is misleading. As I understand, the closed loop implementation is to deliver stimulation when the target muscle was within a window of baseline activation.

Figure 3. Why three baseline tests?

Line 72. "During" rather than "in conjunction"

Line 84. What is ZT12-14

Line 168. Methods are confusing. First, you deliver single pulse spinal stim to produce muscle contraction (line 176). Then the segments were deafferented. (178). Next, the muscle was stimulated to record LFPs to record the antidromic response (latency). Then on lines 182-3 you indicate that you determined the afferent latency. Obviously, this was done before deafferentation. On lines 184-5, you stimulate muscle and record from cortex. This would be the ascending pathway to M1. But, again, this is done before deafferentation. Please re-write for clarity.

Line 197. To clarify, 3 ms is the motoneuron to EMG conduction delay and the afferent latency (line 183).

Line 244. Is normalization baseline correction?
