# Supplementary material for: Paired Associative Stimulation Fails to Induce Plasticity in Freely Behaving Intact Rats
Source: eNeuro. 2020 Mar 19;7(2):ENEURO.0396-19.2020. doi: 10.1523/ENEURO.0396-19.2020 (PMC7113557; doi:10.1523/ENEURO.0396-19.2020)
Supplement: Figure 4-1 — Study design for chronic experiments and a priori randomization order (number within each cell). The final sample sizes for each PAS condition are written to the left. Sessions used in the final dataset are shaded in gray. Download Figure 4-1, DOC file. [file enu-eN-NRS-0396-19-s02.doc]

**Extended Data for Figure 4-1: Study Design for Chronic Experiments**

| **Chronic PAS Experimental Matrix** |  |  |  |  |  |  |  |  |  |  |  |
| --- | --- | --- | --- | --- | --- | --- | --- | --- | --- | --- | --- |
| **Condition** |  | Rat |  |  |  |  |  |  |  |  |  |
|  |  | 1 | 2 | 3 | 4 | 5 | 6 | 7* | 8 | 9 | 10 |
| **ISI Condition** |  |  |  |  |  |  |  |  |  |  |  |
| STDP -23 (N = 7) |  | 14 | 14 | 3 | 1 | 3 | 10 | 3 | 11 | 4 | 1 |
| STDP -33 (N = 8) |  | 9 | 2 | 1 | 11 | 6 | 9 | 4 | 15 | 14 | 15 |
| STDP -48 (N = 7) |  | 7 | 4 | 6 | 6 | 11 | 7 | 13 | 5 | 9 | 6 |
| STDP -15 (N = 5) |  | 15 | 15 | 15 | 15 | 15 | 15 | 2 | 3 | 1 | 14 |
| STDP -10 (N = 6) |  | 12 | 1 | 12 | 14 | 7 | 12 | 8 | 10 | 11 | 10 |
| STDP 0 (N = 6) |  | 1 | 12 | 11 | 12 | 13 | 14 | 5 | 2 | 10 | 8 |
| STDP +6 (N = 7) |  | 2 | 6 | 9 | 7 | 9 | 5 | 7 | 9 | 5 | 13 |
| STDP +10 (N = 8) |  | 6 | 9 | 2 | 4 | 14 | 6 | 15 | 13 | 6 | 5 |
| STDP +12 (N = 7) |  | 10 | 5 | 13 | 10 | 12 | 4 | 1 | 8 | 7 | 12 |
| STDP +15 (N = 7) |  | 8 | 11 | 5 | 3 | 1 | 1 | 12 | 14 | 13 | 9 |
| STDP +25 (N = 6) |  | 13 | 8 | 7 | 13 | 4 | 3 | 14 | 4 | 12 | 11 |
| STDP Timing Control +505 (N = 6) |  | 11 | 3 | 8 | 9 | 2 | 13 | 9 | 12 | 15 | 7 |
|  |  |  |  |  |  |  |  |  |  |  |  |
| **STDP Controls** |  |  |  |  |  |  |  |  |  |  |  |
| Cortex Only Stimulation (Cx) (N = 7) |  | 5 | 7 | 14 | 5 | 10 | 2 | 10 | 1 | 3 | 2 |
| Muscle Only Stimulation (Ms) (N = 6) |  | 3 | 10 | 4 | 8 | 5 | 11 | 11 | 7 | 2 | 3 |
| No Stimulation Control (No) (N = 8) |  | 4 | 13 | 10 | 2 | 8 | 8 | 6 | 6 | 8 | 4 |

* Implant Failure Prevented Data Collection
