# Supplementary material for: Paired Associative Stimulation Fails to Induce Plasticity in Freely Behaving Intact Rats
Source: eNeuro. 2020 Mar 19;7(2):ENEURO.0396-19.2020. doi: 10.1523/ENEURO.0396-19.2020 (PMC7113557; doi:10.1523/ENEURO.0396-19.2020)
Supplement: Figure 4-2 — Muscle distribution. Distribution of muscles used as PAS target (ISI Conditions, Latencies are Stimulation Offsets). *PAS muscle stimulation component was between one EMG electrode and reference. Download Figure 4-2, DOC file. [file enu-eN-NRS-0396-19-s03.doc]

**Extended Data for Figure 4-2: Muscle Distribution**

| Rat/Muscle | *Extensor Carpi Radialis* | *Biceps* | *Trapezius* |
| --- | --- | --- | --- |
| Rat 1 | -10, 0, +6, +10, +12, +15, +25, +505.    -48, -33, -23.    Ms, No, Cx. | --- | --- |
| Rat 2 | --- | --- | -10, 0, +6, +10, +12, +15, +25, +505*.  -15, -23, -33*,  -48*.  Cx, Ms, No. |
| Rat 3 | +6 | --- | +10  -33 |
| Rat 4 | -23  No |  |  |
| Rat 5 | --- | --- | -10, 0, +6, +10, +12, +15, +25, +505.  -15, -23, -33, -48.  Cx, Ms, No. |
| Rat 6 | +6*, +10*, +12*, +15*, +25*.  -33*, -48*.  Cx*, No*. | --- | --- |
| Rat 7 (implant failure before useful data could be collected) | --- | --- | --- |
| Rat 8 | -10*, -0*, +6*, +10*, +12*, +15*, +25*, +505*.  -15*, -23*, -33*,  -48*.  Cx, Ms*, No*. | --- | --- |
| Rat 9  (NB: Manual Range Translation During Closed Loop Due to Heartbeat). | --- | -10*, 0*, +6*, +10*, +12*, +15*, +25*, +505*.  -15*, -23*, -33*, -48*.  Cx*, Ms*, No*. | --- |
| Rat 10 | -10, 0, +6, +10, +12, +15, +25.  -15, -23, -33, -48.  Cx, Ms, No. | --- | --- |
